# Spatiotemporal Dynamics and Climate Change Impacts on Massive Invasive Plants in Henan Province, China

**DOI:** 10.1002/ece3.73870

**Published:** 2026-09-22

**Authors:** Hang Wang, Gaoxiang Cheng, Lvyuan Niu, Yizhen Shao, Fengqin Liu, Yun Chen, Xiangyu Tian, Zhiliang Yuan

**Affiliations:** ^1^ College of Life Sciences Henan Agricultural University Zhengzhou China

**Keywords:** climate change, habitat suitability, Henan province, MaxEnt model, plant invasions

## Abstract

Invasive alien plant species have attracted significant global attention due to their profound ecological disruptions and substantial economic losses. As a critical ecological barrier in the middle and lower reaches of the Yellow River, Henan Province has experienced increasing pressure from biological invasions that jeopardize local biodiversity and ecosystem integrity. In this study, we utilized the species distribution modeling framework to predict the potential geographic distributions of 17 massive invasive plant species under current and future climatic conditions. Our findings indicate that suitable habitats for these massive invasive plants are mainly concentrated in the western and southern regions of Henan. Currently, the high‐risk areas, which are highly suitable for these 17 massive invasive plants, encompass 18.42% of the province's total land area. Under future climate scenarios for 2050, this area is projected to decrease to between 17.27% and 15.99%, with a maximum relative reduction of 13.17% occurring under the highest greenhouse gas emission scenario (SSP585). Furthermore, the results suggest an elevational shift from low to high altitudes in the future among these invasive species, accompanied by a southwestward migration. This research provides a scientific basis for risk assessment and the management of invasive species and offers valuable guidance for biodiversity conservation and ecosystem resilience in the context of global environmental change.

## Graphical abstract

Our findings offer a scientific foundation for the identification, monitoring, and management of malignant invasive alien plants in Henan Province. Given the projected spread of these species, it is crucial to enhance early warning systems and implement targeted regional prevention strategies to minimize their adverse impacts on the environment and economy.

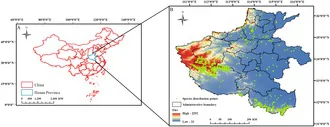

## 1 Introduction

Biological invasions have emerged as a primary driver of biodiversity loss and economic damage (Mori et al. 2026). The impacts of alien species on native biota and human societies are escalating rapidly (Verma et al. 2024). A subset of naturalized species that has overcome dispersal barriers and established self‐sustaining populations far from their original introduction sites is classified as invasive alien plants (Richardson et al. 2001). Currently, nearly one‐fifth of the Earth's surface was threatened by biological invasions (IPBES 2019). Research indicates that future climate change will increase the risk of global invasions worldwide, particularly in Oceania, Africa, and Australia (Adhikari, Lee, Poudel, Hong, and Park 2023; Adhikari, Lee, Poudel, Lee, et al. 2023). Between 1970 and 2017, the global economic cost of these invasions was estimated to exceed USD 26.8 billion annually (Diagne et al. 2021). The damage caused by invasive species has been growing exponentially, and the average annual economic loss is increasing rapidly. In China, the negative effects were particularly acute with over 400 invasive alien plant species (Paini et al. 2016; Hao and Ma 2023). The number of invasive plant species continues to grow at an alarming rate due to the combined effects of international trade (Shrestha and Shrestha 2019). China ranks as the second most economically vulnerable countries (following USA) to invasive alien species. Cumulative costs of invasive alien species reached US$ 3124.66 billion (US$ 2017) (or RMB 21,118.95 billion, with an average of RMB 422.38 billion per year) for total data from 1973 to 2022 and US$ 425.08 billion for robust data, 6.36 times and 3.32 times estimates from Global Invasion Cost Database (InvaCost), respectively. China is currently suffering severe adverse impacts from invasive alien species, with the agricultural sector being hit the hardest (Li et al. 2025; El Jamaai et al. 2026). Among all countries, China may further incur the greatest absolute economic costs resulting from such invasions (Paini et al. 2016). To realize Target 6 of the Convention on Biological Diversity (CBD), China needs to adopt more comprehensive measures to manage invasive alien species. By creating an international greenhouse gas management model that other countries can follow, China's efforts can make a significant contribution to the Kunming‐Montreal 2030 Global Targets of the CBD (Shrestha and Shrestha 2019; Ju et al. 2025).

As a critical transitional zone between the warm temperate and continental monsoon climates, Henan Province plays a key role in ecological and agricultural corridor in China. With spanning approximately 167,000 km^2^ from middle to lower reaches of the Yellow River, Henan present a diverse topography, ranging from the rugged Taihang and Funiu Mountains to the expansive Huang‐Huai‐Hai alluvial plain (Ren et al. 2022). The region has an annual average precipitation of 500–1000 mm, and the climate distribution shows significant spatial heterogeneity (Ren et al. 2022). These features provide advantages for agricultural production; moreover, they provide favorable conditions for the colonization and spread of invasive species (Öztop and Farooq 2025). Especially with the rapid industrialization and urbanization, the pace of ecological restoration has lagged behind economic development, leaving native habitats vulnerable to degradation and biodiversity decline (Fu and Zhang 2021).

The invasive alien plant species in Henan Province is increasingly associated with international trade, climate change, and anthropogenic activities (Bradley et al. 2010). Numerous invasive plants have been introduced, expanded, and caused significant threats to local ecosystems. Simultaneously, global warming provides invasive plants with a competitive advantage over native flora, allowing them to shift niches and colonize higher latitudes or elevations that were previously climatically restrictive regions (Walther et al. 2009). These invasive plants destabilize ecosystem functions by altering nutrient cycles, acidifying soils, and outcompeting native species for resources (Biban et al. 2025). Despite these escalating risks, the dynamics of invasive plants under climate change within this region remain limited, and region‐specific management strategies are still lacking (Ren et al. 2022). A unified consensus has yet to be reached as to whether future climate shifts will cause an expansion or contraction of invasive ranges, making it difficult to formulate localized management strategies (Ziska et al. 2019; Barbet‐Massin et al. 2013; Morales‐Castilla et al. 2015). Some studies suggested that climate change may drive mountain species to migrate upslope at an average rate of approximately 11 m per decade to track optimal climatic conditions (Chen et al. 2011). For a major grain‐producing province like Henan, the potential encroachment of massive invasive plants into farmlands poses a direct threat to national food security, yet priority areas for prevention and control have not been clearly identified.

Species distribution modeling (SDM) has been widely employed to assess the risk of invasive alien species (Higgins and Richardson 2014). Species distribution modeling is a method that combines environmental data with known species occurrence locations to predict and explain species distribution in geographic space. The maximum entropy model (MaxEnt) is frequently used among various available modeling techniques, especially when dealing with limited or incomplete distribution data, as it yields reliable results (Xu et al. 2019). For example, Fu et al. (2022) successfully employed the MaxEnt model to predict the suitable area of *Alternanthera philoxeroides* in China, providing a theoretical basis for preventing its spread.

Although invasive alien plants have significantly affected agricultural production and the ecological environment in Henan Province, systematic research on the specific distribution characteristics, influencing factors, and future distribution patterns of invasive alien plants is still insufficient. This study aims to predict the suitable habitat distribution patterns of massive invasive plants in Henan Province under current and future climate scenarios through the MaxEnt model, based on species occurrence records and environmental variables. Through this study, we will receive the current distribution of these massive invasive plants, predict shifts in suitable habitats under three distinct future climate change scenarios, and identify priority zones for ecological monitoring and management. By clarifying the spatial dynamics of plant invasions, this research not only helps to reveal the invasion mechanism, but also provides a scientific basis for regional ecological security protection and invasion management.

## 2 Materials and Methods

### 2.1 Sources of Invasive Alien Plant Species

**FIGURE 1:**
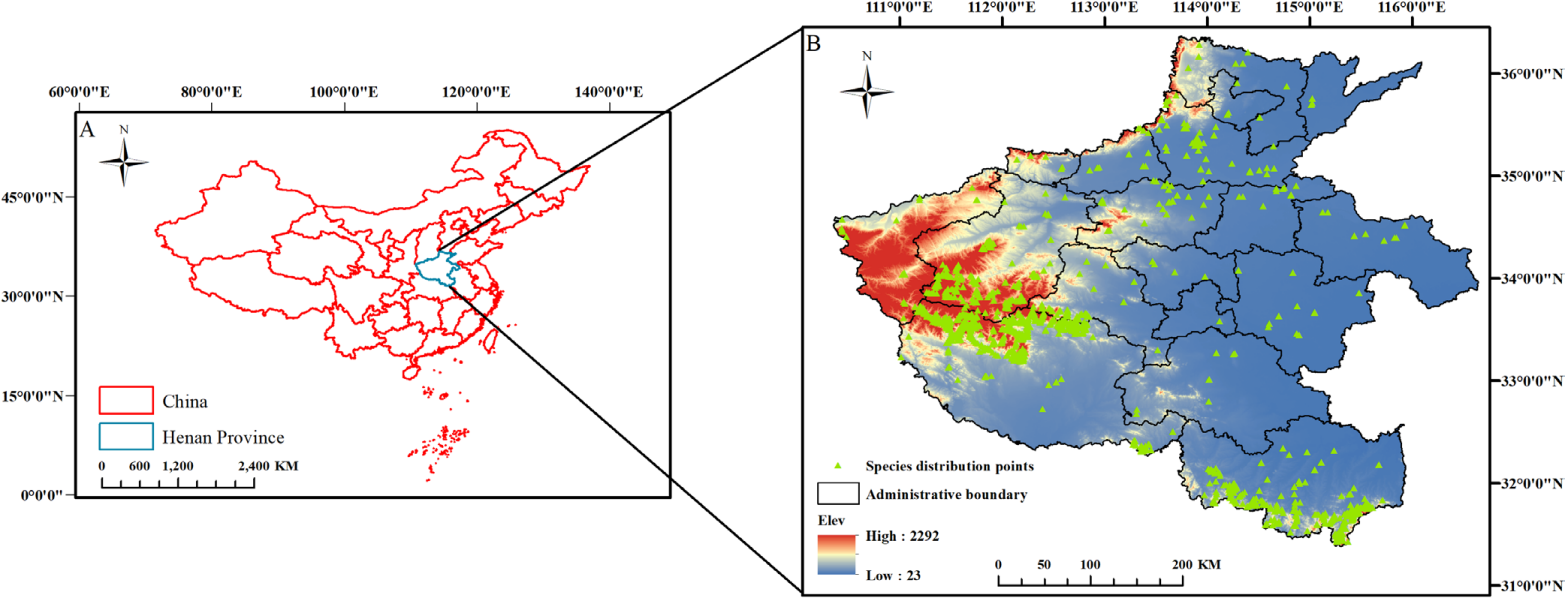
(A) Geographic location of Henan Province in China. (B) Elevation map of Henan Province and the occurrence points of 17 massive invasive alien plants. The map was made based on the standard map No. GS(2019)1822 downloaded from the standard map service website of the State Administration of Surveying, Mapping and Geographic Information, and the base map was not modified.

The massive invasive alien plant species (i.e., those that have caused substantial economic or ecological losses and severe impacts at the national level, and whose invasion range spans more than one physiographic region—Xiaoling et al. 2014) analyzed in this study were identified based on field surveys conducted by our research team across various counties in Henan Province from 2023 to the present. Additional data were obtained from authoritative floras, including *Flora of China*, *Flora of Henan*, as well as from peer‐reviewed articles, books, and databases such as CNKI (https://www.cnki.net), the National Plant Specimen Resource Center (NPSRC, https://www.cvh.ac.cn), and the China Invasive Alien Species Information System (IASC, https://www.plantplus.cn/ias/). In total, 34 species were documented. To obtain occurrence records of massive invasive alien plant species in Henan Province, we combined field surveys with data from online databases, including the Global Biodiversity Information Facility (GBIF) and the Chinese Virtual Herbarium (CVH). To minimize the impact of sampling bias and ensure the accuracy of species distribution model predictions (Dubos et al. 2022), we filtered the 34 species occurrence records using ArcGIS (v. 10.8; Esri, Redlands, California, USA). Duplicate records were removed, and a 1 km^2^ buffer was applied around each point. Within each buffer, only one occurrence record per species was retained to avoid spatial autocorrelation (Sorbe et al. 2023). To ensure modeling reliability, we included only species with more than 10 occurrence records within Henan Province (Ye et al. 2021). After filtering, a total of 2822 occurrence records representing 17 species were retained for species distribution modeling (Figure 1).

Their life forms were categorized as annual or biennial herbaceous plants, perennial herbaceous plants, perennial aquatic herbaceous plants, shrubs, annual or perennial herbaceous plants, and lianas. Habitats were classified as aquatic, terrestrial, or amphibious. Introduction pathways were divided into intentional and unintentional introductions. Species origins were assigned according to the World Geographical Scheme for Recording Plant Distributions (WGSRPD) and grouped into five continental regions: Europe, Africa, Asia, North America, and South America (Table S1) (Brummitt et al. 2001). For species with multiple geographic origins, each origin was counted separately (Chu et al. 2024).

### 2.2 Environmental Variables

A total of 39 environmental variables were used in this study to model species distributions. These included 19 bioclimatic variables (bio1–bio19), 16 top soil (0–30 cm) variables, 3 topographical variables (elevation, slope, aspect), and one anthropogenic variable—human footprint (Table S2). The elevation and bioclimatic variables were obtained at a spatial resolution of 30 arc‐seconds (approximately 1 km) from the WorldClimate database (WorldClim v.2.1, https://www.worldclim.org/). The slope and aspect were derived from elevation data using the Spatial Analyst tools in ArcGIS. The top soil variables were obtained from the National Cryosphere Desert Data Center (http://www.ncdc.ac.cn), with data for China sourced from the Second National Land Survey, provided by the Nanjing Institute of Soil Science, Chinese Academy of Sciences, at a scale of 1:1,000,000 (Fischer et al. 2008). The human footprint variable integrates both remote sensing and bottom‐up survey data from the years 1993 and 2009, reflecting direct and indirect human pressures on the environment. It comprises eight components: (1) extent of built environments, (2) cropland, (3) pasture, (4) human population density, (5) nighttime lights, (6) railways, (7) roads, and (8) navigable waterways. These components were weighted based on the relative intensity of human pressure, following the methodology proposed by Sanderson et al. and aggregated to produce a standardized global human footprint index for all non‐Antarctic land areas (Venter et al. 2016). To obtain future climate data, we selected three Shared Socioeconomic Pathways (SSP126, SSP245 and SSP585) for the year 2050, representing low concentration, medium concentration, and high concentration emission greenhouse gas scenarios, respectively (O'Neill et al. 2014). The Global Climate Model (GCM) used in this study was BCC‐CSM2‐MR (Beijing Climate Center Climate System Model, Medium‐resolution—Wu, Lu, et al. 2019), which has been widely applied in studies conducted in China and the Himalayan region. In this study, we assumed that elevation, slope, aspect, top soil variables, and human footprint would remain constant through 2050. While this assumption simplifies the modeling process, it inevitably introduces some uncertainty into the projections (Chu et al. 2024; Qin et al. 2024).

### 2.3 MaxEnt Model Construction

We used MaxEnt (v.3.4.3) to predict the geographic distribution of massive invasive alien plant species (Phillips et al. 2006). For each species, 75% of the occurrence records were randomly selected as training data, while the remaining 25% served as testing data (Lu et al. 2021). We employed a random seed with the replicated run‐type set as “bootstrap” and conducted 10 replicates. Additionally, we generated response curves and performed jackknife analysis to assess variable importance (Phillips et al. 2006). The default parameters were used for all other settings. According to the average results of 10 operations of the initial model, the environmental variables whose contribution rate was less than 1% were removed. To prevent overfitting of the model resulting from multicollinearity among environmental variables, which can impact the accuracy of the results (Yi et al. 2016), the Pearson correlation coefficients between 39 environmental variables were calculated in this study (Table S3). Based on the contribution rate or permutation importance, one of the pairs of variables with high correlation was removed (|*r*| > 0.8) (Table S4) (Lu et al. 2021). The screened environmental variables were used for the final modeling, and the variables corresponding to each species are provided in Table S4 (Lu et al. 2021).

We used ArcGIS to convert the continuous raster pixel values predicted for individual species into binary values (0 for unsuitable, 1 for suitable) (Urbani et al. 2015). Then, using the Raster Calculator in ArcGIS, we overlaid the binary habitat suitability maps of 17 species to generate a species richness distribution map (Figure 6A). Species richness is the simplest way to describe biotic community and regional diversity (Magurran 1988). It refers to the number of species in an area, biotic community, or ecosystem (Riosmena‐Rodriguez et al. 2016). Elevation and species richness values (ranging from 0 to 13) were extracted from species occurrence points to create a line graph (Figure 6B).

### 2.4 Model Accuracy Evaluation

The area under the receiver operating characteristic curve (AUC) is the most commonly used diagnostic metric in species distribution modeling (SDM) (Porfirio et al. 2014). Generally, when AUC > 0.9, the model is considered to have excellent performance and is suitable for further analysis (Chen et al. 2023). Habitat Suitability Index (HSI) models are widely used in wildlife management to assess species‐environment relationships and inform conservation strategies. Initially, the United States Fish and Wildlife Service (USFWS), in cooperation with the US Army Corps of Engineers and other federal agencies, developed the habitat evaluation procedures (HEP) system to determine the quality and quantity of habitat for fish and wildlife (Zhang, Zhao, et al. 2025). Habitat suitability maps were generated in ArcGIS, with the habitat suitability index ranging from 0 to 1. Higher values indicate a greater likelihood of the presence of invasive alien plants. HSI values were classified into four categories: unsuitable (0–0.2), low suitability (0.2–0.4), moderate suitability (0.4–0.6), and high suitability (0.6–1) (Chu et al. 2024; Wu, Lu, et al. 2019). Using ArcGIS, we overlaid the suitable habitat distribution maps and richness distribution maps of 17 massive invasive alien plant species in Henan Province. The SDMtoolbox 2.0 was employed to calculate the suitable habitat area under current and future climate scenarios, as well as future contraction and expansion areas, and to assess the spatial shifts of suitable habitats. In this study, grid cells with HSI values greater than or equal to 0.4 were considered suitable potential geographic distribution areas for massive invasive alien plants (Ye et al. 2021).

## 3 Results

### 3.1 Taxonomy and Species Composition of Massive Invasive Alien Plants in Henan Province

**FIGURE 2:**
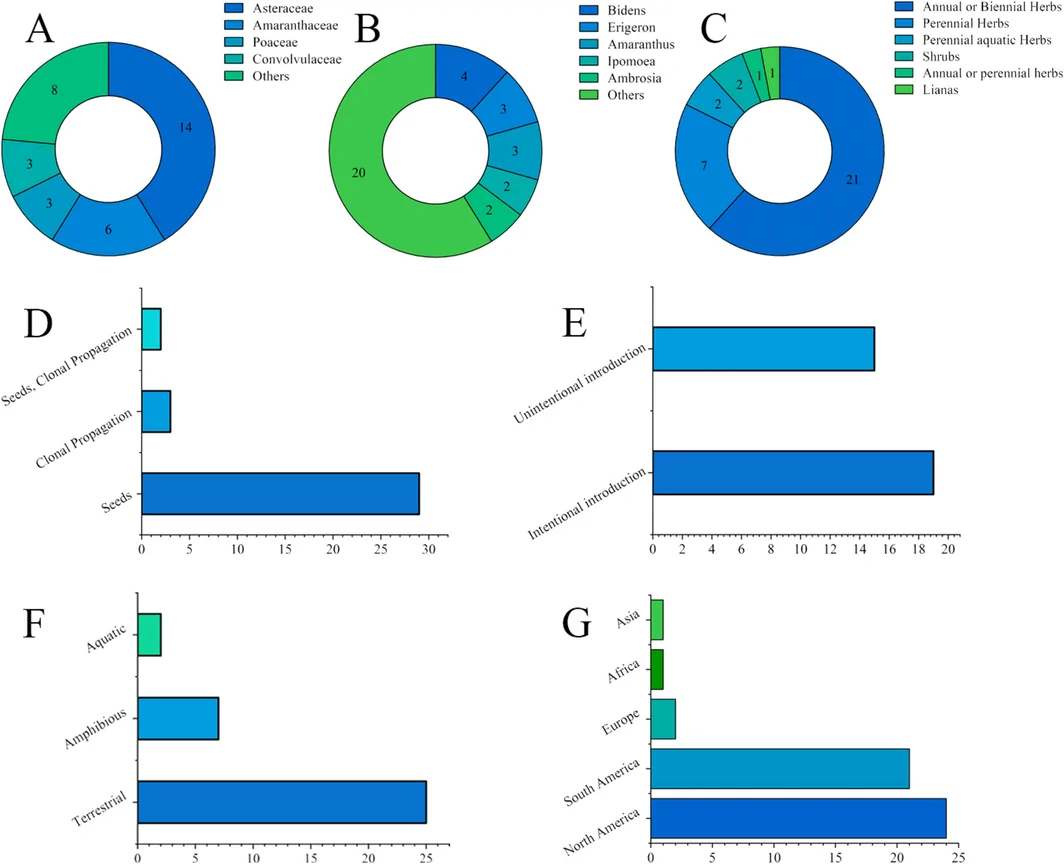
Characteristics of massive invasive alien plants in Henan Province. (A) Identified plant families; (B) identified plant genera; (C) life forms; (D) reproductive characteristics; (E) pathways of introduction; (F) habitats; (G) origin.

A total of 37 massive invasive alien plant species were recorded in this study, all of which are Angiosperms, representing 25 plant genera in 12 families. Among these, the family *Asteraceae* was the most frequently represented, with 14 species, accounting for 41.18% of the total. This was followed by Amaranthaceae with 6 species (17.65%), and both Poaceae and Convolvulaceae with 3 species each (8.82%) (Figure 2A). At the genus level, *Bidens* L. was the most frequently represented, with 4 species (11.76%), followed by *Erigeron* L. and *Amaranthus* L., each with 3 species (8.82%) (Figure 2B).

Among the different life forms of massive invasive alien plants in Henan Province, annual or biennial herbaceous plants were the most common (21 species), followed by perennial herbaceous plants (7 species), and both perennial aquatic herbaceous plants and shrubs had 2 species each (Figure 2C). Most of these invasive plants reproduce by seeds (29 species), while 3 species reproduce clonally, and 2 species utilize both clonal and seed reproduction (Figure 2D). Intentional introduction was the main pathway (19 species), while 15 species were unintentionally introduced (Figure 2E). The habitats are primarily terrestrial (25 species), followed by amphibious (7 species), with the fewest being aquatic (2 species) (Figure 2F). In terms of geographic origin, species from North America were the most frequent (24 occurrences), followed by South America (21 occurrences), and Europe (2 occurrences) (Figure 2G).

### 3.2 Performance of the MaxEnt Model

The results of the MaxEnt model showed that the AUC values for each species at different time periods ranged from 0.905 to 0.972 (Table 1), indicating that the constructed MaxEnt models performed well. These simulation results are reliable for predicting and analyzing the potential geographic distribution of the species.

**TABLE 1:** AUC values of the MaxEnt model for each period and climate scenario.

Species	Current	2050s		
		SSP126	SSP245	SSP585
*Bidens alba* (L.) DC	0.972	0.968	0.97	0.966
*Phytolacca americana* L.	0.954	0.951	0.951	0.955
*Amaranthus spinosus* L.	0.964	0.961	0.969	0.965
*Bidens frondosa* L.	0.936	0.952	0.946	0.945
*Amaranthus retroflexus* L.	0.925	0.923	0.939	0.94
*Bidens pilosa* L.	0.934	0.938	0.938	0.932
*Ageratum conyzoides* L.	0.959	0.952	0.962	0.967
*Bidens bipinnata* L.	0.962	0.962	0.958	0.962
*Celosia argentea* L.	0.952	0.95	0.956	0.95
*Erigeron sumatrensis* Retz.	0.964	0.96	0.969	0.954
*Dysphania ambrosioides* (L.) Mosyakin & Clemants	0.96	0.969	0.97	0.974
*Ambrosia artemisiifolia* L.	0.953	0.957	0.966	0.971
*Alternanthera philoxeroides* (Mart.) Griseb.	0.958	0.938	0.946	0.948
*Erigeron canadensis* L.	0.905	0.912	0.906	0.909
*Erigeron annuus* (L.) Pers	0.933	0.936	0.934	0.94
*Ipomoea purpurea* (L.) Roth	0.923	0.929	0.931	0.918
*Symphyotrichum subulatum* (Michx.) G. L. Nesom	0.944	0.959	0.944	0.960

### 3.3 Predicted Current and Future Potential Distribution Area

**FIGURE 3:**
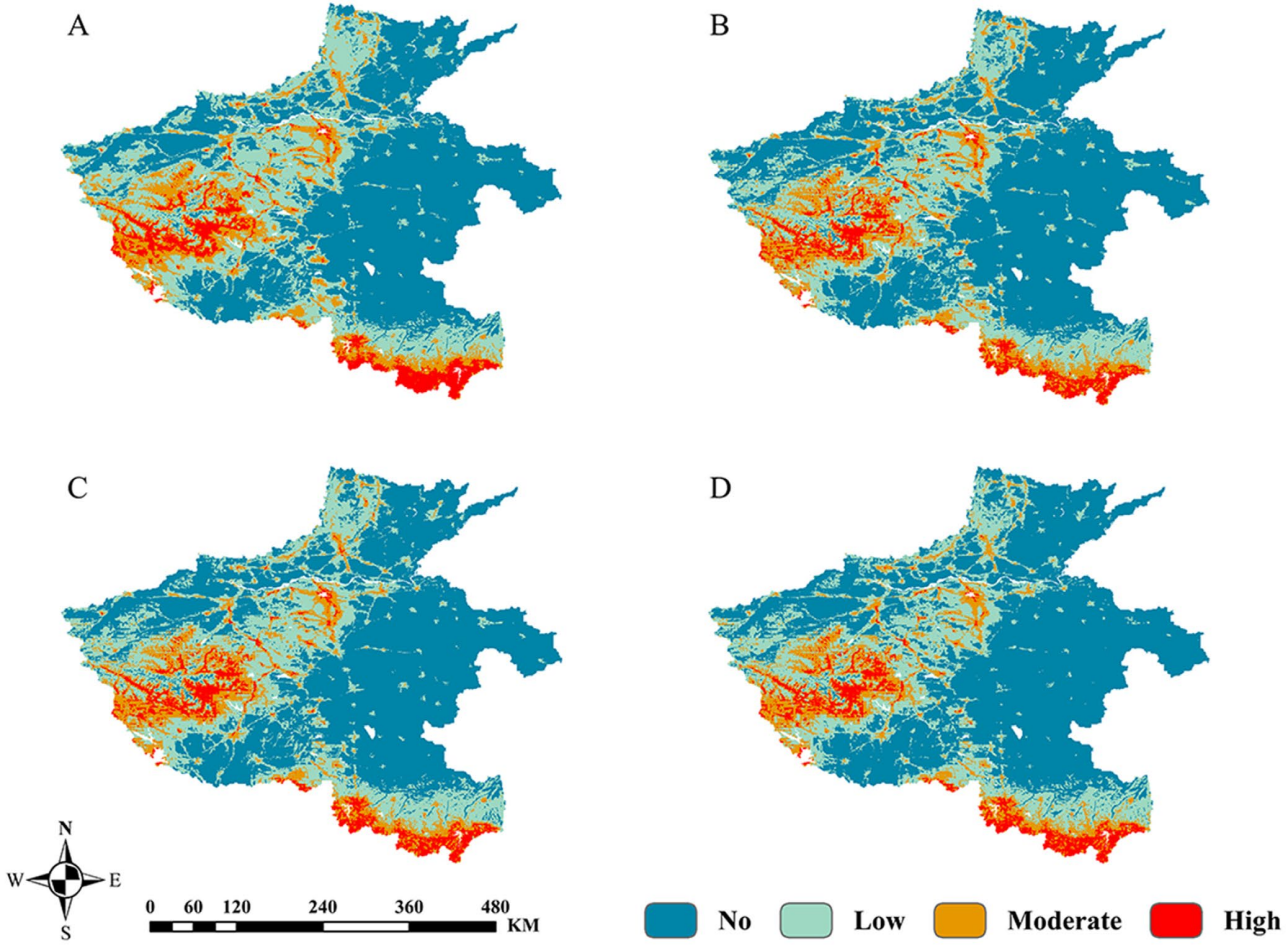
Current and future habitat suitability simulation of the potential distribution area for 17 massive invasive alien plants in Henan Province. (A) Current; (B) SSP126‐2050; (C) SSP245‐2050; (D) SSP585‐2050.

The potential suitable habitats of the 17 massive invasive alien plant species are mainly concentrated in the western, southern, and to a lesser extent, northern regions of Henan Province (Figure 3) (Qin et al. 2024; Yang et al. 2025). Currently, these species potential distribution areas cover 18.42% of the total area of Henan Province (Table 2). With increasing greenhouse gas emissions, this percentage is projected to decline by 2050, from 17.27% to 15.99% (Table 2). Under the SSP126 scenario, the relative reduction is minimal at 6.25%, while under the SSP585 scenario, the relative reduction is the most severe at 13.17% (Table 2). Currently, *Erigeron canadensis* , *Erigeron annuus*, and *Ipomoea purpurea* have the largest potential distribution areas in Henan Province, with estimated areas of 20,640, 16,970, and 15,000 km^2^, accounting for 13.16%, 10.82%, and 9.55% of the province's total area, respectively (Table 2). In contrast, *Amaranthus spinosus* , *Erigeron sumatrensis*, and *Dysphania ambrosioides* exhibit the smallest potential distributions, with areas of 5560, 5610, and 6030 km^2^, representing 3.54%, 3.58%, and 3.84%, respectively (Table 2).

**TABLE 2:** Current and future potential distribution area of 17 massive invasive alien plants.

Species	Potential distribution area (×10^4^ km^2^)								Proportion of potential distribution area (%)			
	Moderate				High							
	Current	2050‐SSP126	2050‐SSP245	2050‐SSP585	Current	2050‐SSP126	2050‐SSP245	2050‐SSP585	Current	2050‐SSP126	2050‐SSP245	2050‐SSP585
*Bidens alba* (L.) DC	0.562	0.668 (+18.91%)	0.578 (+2.79)	0.654 (+16.43)	0.458	0.413 (−9.98%)	0.462 (+0.70%)	0.451 (−1.52%)	6.5	6.89 (+5.93%)	6.62 (+1.85%)	7.05 (+8.37%)
*Phytolacca americana* L.	0.512	0.508 (−0.80%)	0.452 (−11.59%)	0.395 (−22.77%)	0.502	0.528 (+5.24%)	0.441 (−12.04%)	0.476 (−5.22%)	6.46	6.6 (+2.19%)	5.7 (−11.81%)	5.55 (−14.08%)
*Amaranthus spinosus* L.	0.379	0.473 (+24.78%)	0.306 (−19.31%)	0.286 (−24.51%)	0.177	0.238 (+34.62%)	0.201 (+13.95%)	0.213 (+20.71%)	3.54	4.53 (+27.91%)	3.23 (−8.74%)	3.18 (−10.14%)
*Bidens frondosa* L.	0.822	0.628 (−23.57%)	0.669 (−18.65%)	1.321 (+60.68%)	0.605	0.399 (−34.13%)	0.317 (−47.67%)	0.542 (−10.48%)	9.1	6.54 (−28.05%)	6.28 (−30.96%)	11.87 (+30.51%)
*Amaranthus retroflexus* L.	0.554	0.591 (+6.68%)	0.551 (−0.61%)	0.49 (−11.59%)	0.629	0.573 (−8.99%)	0.504 (−19.91%)	0.423 (−32.71%)	7.54	7.42 (−1.65%)	6.72 (−10.87%)	5.82 (−22.82%)
*Bidens pilosa* L.	0.702	0.594 (−15.35%)	0.582 (−17.11%)	0.639 (−9.00%)	0.783	0.586 (−25.14%)	0.598 (−23.69%)	0.628 (−19.89%)	9.47	7.52 (−20.52%)	7.52 (−20.58%)	8.07 (−14.75%)
*Ageratum conyzoides* L.	0.531	0.546 (+2.85%)	0.171 (−67.74%)	0.44 (−17.12%)	0.075	0.034 (−54.97%)	0.006 (−91.92%)	0.034 (−54.32%)	3.86	3.69 (−4.29%)	1.13 (−70.73%)	3.02 (−21.71%)
*Bidens bipinnata* L.	0.421	0.355 (−15.75%)	0.463 (+10.07%)	0.455 (+8.24%)	0.290	0.23 (−20.65%)	0.326 (+12.51%)	0.33 (+13.75%)	4.53	3.72 (−17.75%)	5.03 (−11.06%)	5 (+10.49%)
*Celosia argentea* L.	0.474	0.505 (+6.64%)	0.407 (−14.10%)	0.427 (−9.92%)	0.340	0.319 (−6.19%)	0.268 (−21.23%)	0.3 (−11.69%)	5.19	5.25 (+1.28%)	4.3 (−17.08%)	4.63 (−10.66%)
*Erigeron sumatrensis* Retz.	0.481	0.345 (−28.26%)	0.303 (−37.09%)	0.209 (−56.56%)	0.080	0.116 (+43.66%)	0.025 (−68.68%)	0.00049 (−99.40%)	3.58	2.94 (−17.96%)	2.09 (−41.62%)	1.34 (−62.69%)
*Dysphania ambrosioides* (L.) Mosyakin & Clemants	0.346	0.249 (−28.00%)	0.229 (−33.90%)	0.225 (−34.85%)	0.257	0.184 (−28.25%)	0.21 (−18.34%)	0.189 (−26.49%)	3.84	2.76 (−28.11%)	2.79 (−27.28%)	2.64 (−31.29%)
*Ambrosia artemisiifolia* L.	0.804	0.8 (−0.46%)	0.705 (−12.33%)	0.626 (−22.16%)	0.469	0.258 (−44.86%)	0.33 (−29.48%)	0.233 (−50.17%)	8.11	6.75 (−16.81%)	6.6 (−18.64%)	5.48 (−32.47%)
*Alternanthera philoxeroides* (Mart.) Griseb.	0.350	0.444 (+27.00%)	0.463 (+32.22%)	0.453 (+29.38%)	0.412	0.504 (+22.27%)	0.397 (−3.76%)	0.399 (−3.17%)	4.86	6.04 (+24.44%)	5.47 (+12.76%)	5.43 (+11.78%)
*Erigeron canadensis* L.	0.840	0.76 (−9.56%)	0.802 (−4.49%)	0.784 (−6.68%)	1.224	1.102 (−9.98%)	1.089 (−11.09%)	1.02 (−16.72%)	13.16	11.86 (−9.81%)	12.05 (−8.4%)	11.49 (−12.63%)
*Erigeron annuus* (L.) Pers	0.832	0.648 (−22.12%)	0.742 (−10.88%)	1.123 (+34.91%)	0.865	0.651 (−24.73%)	0.717 (−17.17%)	0.668 (−22.78%)	10.82	8.28 (−23.45%)	9.29 (−14.08%)	11.41 (+5.51%)
*Ipomoea purpurea* (L.) Roth	0.826	0.653 (−20.88%)	0.772 (−6.51%)	0.801 (−2.94%)	0.674	0.503 (−25.29%)	0.645 (−4.27%)	0.675 (+0.21%)	9.55	7.37 (−22.86%)	9.03 (−5.5%)	9.41 (−1.52%)
*Symphyotrichum subulatum* (Michx.) G. L. Nesom	0.584	0.359 (−38.50%)	1.012 (+73.21%)	0.188 (−67.78%)	0.175	0.176 (+0.91%)	0.534 (+205.64%)	0.067 (−61.42%)	4.84	3.41 (−29.42%)	9.85 (+103.7%)	1.63 (−66.32%)
Total	1.975	1.933 (−2.13%)	1.827 (−7.53%)	1.74 (−11.92%)	0.915	0.776 (−15.13%)	0.789 (−13.78%)	0.77 (−15.86%)	18.42	17.27 (−6.25%)	16.67 (−9.51%)	15.99 (−13.17%)

By 2050, the potential suitable habitats for most massive invasive alien plant species in Henan Province are projected to shrink under different climate scenarios. The suitable habitat areas of *Amaranthus retroflexus* , *Erigeron sumatrensis*, and *Ambrosia artemisiifolia* are expected to decline progressively with increasing greenhouse gas emissions, by 1.65%–22.82%, 17.96%–62.69%, and 16.81%–32.47%, respectively (Table 2). Under the SSP126 scenario, *Bidens pilosa* and *I. purpurea* show the greatest reductions in suitable area (20.52% and 22.86%, respectively), whereas under the SSP585 scenario, the reductions are minimal (14.75% and 1.52%, respectively) (Table 2). For *Ageratum conyzoides* , the smallest reduction occurs under SSP126 (4.29%), while the greatest reduction occurs under SSP245 (70.73%) (Table 2). *D. ambrosioides* and *E. canadensis* show moderate reductions under SSP126 and SSP245 (28.11%, 9.81% and 27.28%, 8.4%, respectively), with maximum reductions under SSP585 (31.29% and 12.63%) (Table 2). *E. annuus* , *Bidens bipinnata* , and *Bidens frondosa* show reductions under SSP126 and SSP245 (23.45%, 17.75%, 28.05% and 14.08%, 11.06%, 30.96%, respectively), but increases under SSP585 (5.51%, 10.49%, and 30.51%) (Table 2).

*Phytolacca americana* , *A. spinosus* , and *Celosia argentea* exhibit increases under SSP126 (2.19%, 27.91%, and 1.28%, respectively), but decreases under SSP245 (11.81%, 8.74%, and 17.08%) and SSP585 (14.08%, 10.14%, and 10.66%) (Table 2). Notably, *Symphyotrichum subulatum* is projected to expand significantly under SSP245, with a 103.7% increase in suitable habitat, but will decline under SSP126 and SSP585 by 29.42% and 66.32%, respectively (Table 2).

Importantly, both *Alternanthera philoxeroides* and *Bidens alba* are projected to experience increases in suitable habitat under all three climate scenarios by 2050. The area suitable for *A. philoxeroides* is expected to increase by 11.78%–24.44%, and that for *B. alba* by 1.85%–8.37%, with no observed contraction under any scenario (Table 2).

### 3.4 Predicted Changes in the Potential Distribution Area From the Present to 2050

**FIGURE 4:**
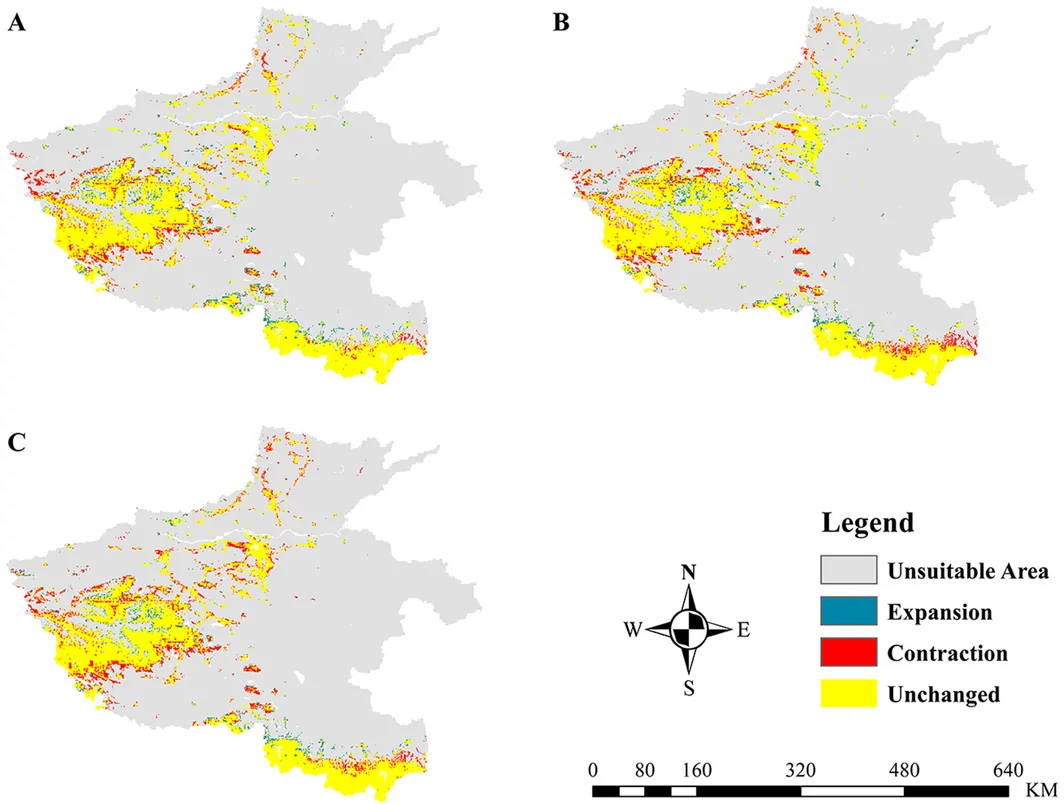
Changes in the predicted potential habitat of 17 massive invasive alien plants under different climate scenarios in 2050. (A) SSP126‐2050; (B) SSP245‐2050; (C) SSP585‐2050.

Compared to the current period, by 2050, potential habitats under the three climate scenarios are projected to undergo varying degrees of contraction (Figure 4). The contraction area increases with rising greenhouse gas emissions, ranging from 3840 to 5270 km^2^, with 13.29%–18.24% of currently suitable areas expected to become unsuitable (Table 3).

**TABLE 3:** Changes in the predicted potential suitable area of 17 massive invasive alien plants under different climate scenarios in 2050 compared to the current climatic condition.

Climate scenario	Area (×10^4^ km^2^)				
	Unsuitable area	Contraction	Expansion	Unchanged suitable area	Total suitable area
Current	12.802	—	—	2.890	2.890
2050‐SSP126	12.656	0.384 (13.29%)	0.203 (7.04%)	2.506	2.710 (−6.25%)
2050‐SSP245	12.633	0.444 (15.36%)	0.169 (5.85%)	2.446	2.615 (−9.51%)
2050‐SSP585	12.656	0.527 (18.24%)	0.147 (5.07%)	2.363	2.51 (−13.17%)

In contrast, habitat expansion is relatively limited and decreases as emissions rise, ranging from 1470 to 2030 km^2^, with 5.07%–7.04% of currently unsuitable areas predicted to become suitable. Most regions are expected to remain stable, with stable areas ranging from 23,630 to 25,060 km^2^(Table 3).

Additionally, in the western part of Henan Province, where elevation ranges from 0 to 2292 m, suitable habitats for invasive alien plants show a pattern of contraction in low‐altitude areas and expansion in high‐altitude areas (Figure 4). This indicates a trend of massive invasive plant shifting from lower to higher elevations. In the southern mountainous region (elevation 0–1493 m), invasive plants primarily show a contraction trend in low‐altitude areas, while high‐altitude areas have already been colonized (Figure 4). As a result, no further expansion of suitable habitat is expected in those high‐altitude zones.

### 3.5 Migration Trend of Geometric Center in Suitable Habitats of Massive Invasive Alien Plants in Henan Province

**FIGURE 5:**
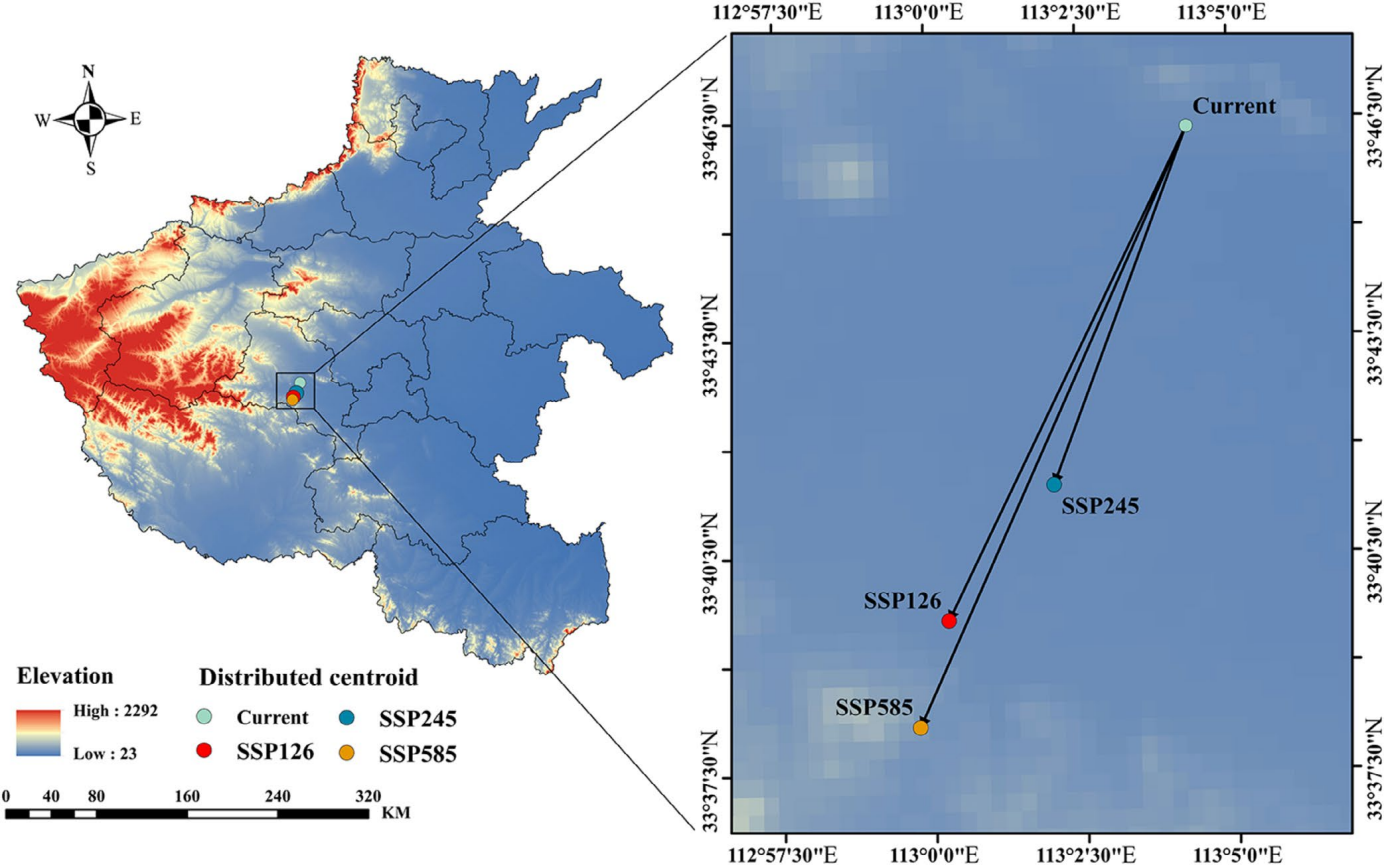
Geometric center of 17 massive invasive alien plants under different climate change scenarios.

In the current period, the geometric center of suitable habitats for massive invasive alien plants is located in Xinhua District, Pingdingshan City (113.071971° E, 33.773064° N) (Figure 5). By 2050, under all three climate scenarios, the geometric center is projected to shift southwestward to Lushan County in Henan Province (Figure 5). The migration distance is approximately 14.033 km under the SSP126 scenario, about 9.774 km under SSP245 (the shortest shift), and around 16.815 km under SSP585 (the farthest shift) (Figure 5).

### 3.6 Current Species Richness Potential Distribution

**FIGURE 6:**
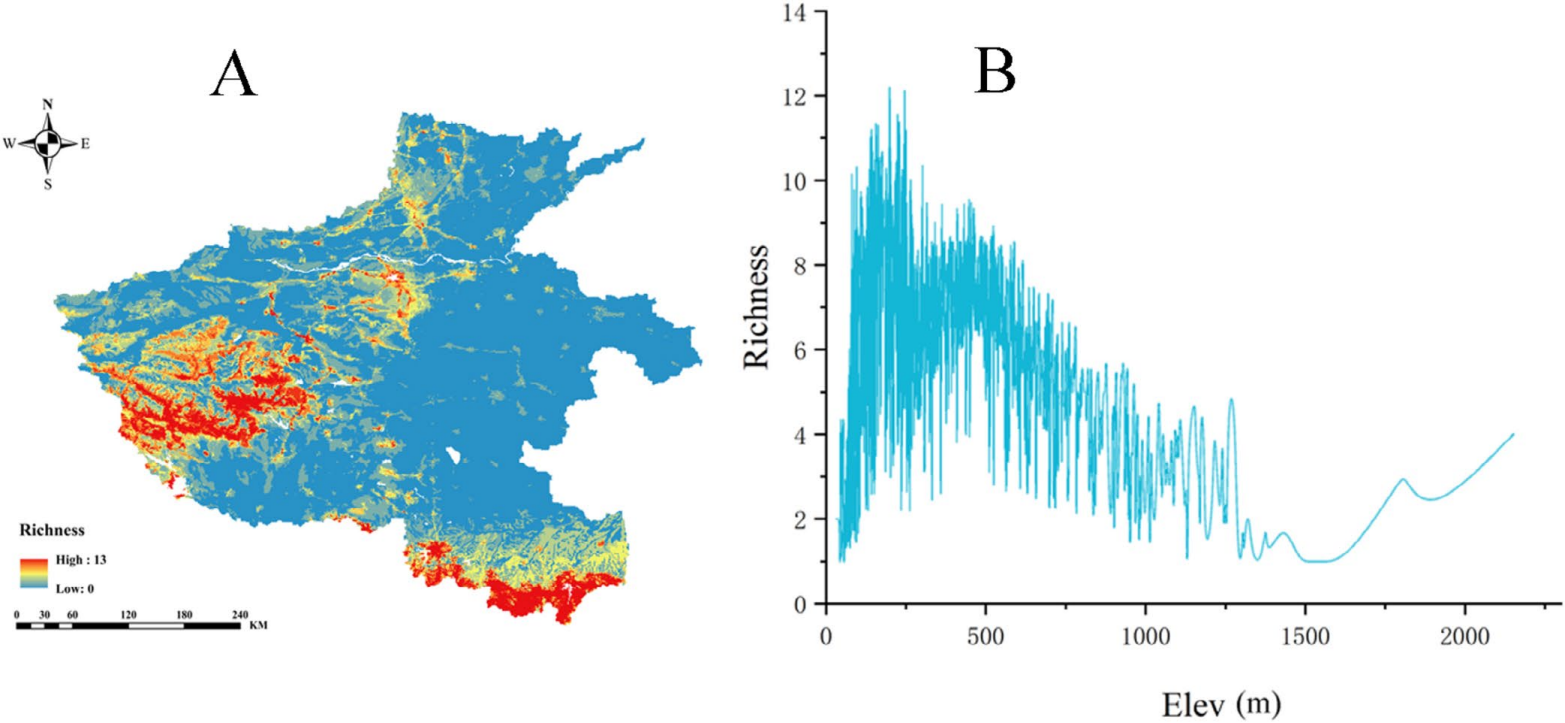
(A) Species richness distribution map of 17 massive invasive alien plants in Henan Province; (B) variation in species richness of 17 massive invasive alien plants along an elevation gradient in Henan Province.

Regions with lower richness values are primarily concentrated in Zhengzhou (the provincial capital), Xinxiang city, Kaifeng city, and the Taihang Mountains (Figure 6A). Higher richness areas are mainly located in the mountainous regions of western Henan—such as the Xiong'er Mountains, Funiu Mountains, and Waifang Mountains—as well as in the Dabie Mountains, Jigong Mountains, and Tongbai Mountains in the south (Figure 6A).

In low‐elevation regions (0–250 m), species richness tends to increase with elevation. In mid‐elevation zones (250–1500 m), richness shows a declining trend. In high‐elevation areas (> 1500 m), species richness increases again (Figure 6B). For the aforementioned high‐richness areas, future efforts should focus on strengthening supervision and preventing further spread of massive invasive alien plants. In low‐richness areas, early monitoring and rapid response mechanisms should be enhanced to prevent the further expansion and establishment of invasive species, thereby mitigating invasion risks at the source.

## 4 Discussion

The dominance of Asteraceae is the most representative family. Asteraceae is one of the largest plant families globally, comprising approximately 1620 genera and 23,600 species (Agbede et al. 2023). Asteraceae species are particularly prominent among invasive plants in China (Zhang, Gu, et al. 2025), and numerous studies have highlighted their significance in biological invasions, often attributed to their high reproductive capacity (Chu et al. 2024; Weidlich et al. 2020; Huang et al. 2025).

In terms of life forms, the majority of massive alien invasive plants in Henan Province are annual or biennial herbs and perennial herbs, consistent with findings from other regions (Chu et al. 2024; Wagh and Jain 2018; Wang et al. 2022). It has been suggested that herbaceous plants are a common group among invasive species (Chu et al. 2024), and that herbaceous plants are generally more likely to succeed in invasions than woody species (Weber 2017). In particular, annual invasive herbs can outcompete native perennials during the seedling stage, potentially leading to the decline or extinction of native species (Humphrey and Schupp 2004; Graeiela Melgoza and Tausch 1990). The predominance of seed‐based reproduction further facilitates long‐distance dispersal and rapid colonization (McConkey et al. 2012).

Regarding pathways of introduction, the majority of massive invasive plants in Henan were intentionally introduced. Intentional introductions are usually associated with economic uses (van Kleunen et al. 2020). Species with economic value are 18 times more likely to naturalize globally, and those with larger native ranges are more likely to be utilized by humans, thereby accelerating their invasion success (Guo, Pyšek, et al. 2024). North America was the most frequent origin for massive invasive plants in Henan. This pattern may be due to similarities in environmental and climatic conditions between these regions and Henan (McDougall et al. 2005), making species from the Americas more likely to establish and spread successfully.

Among the three batches of alien invasive plant lists published by China's Ministry of Ecology and Environment, 20 of the 29 listed massive invasive plants have been recorded in Henan Province, indicating their strong invasive potential and capacity for rapid spread within China (Chu et al. 2024). Specifically, among the nine species in the first batch, *Alternanthera philoxeroides* , *Ambrosia artemisiifolia* , *Lolium temulentum* , *Spartina alterniflora* , and *Eichhornia crassipes* are present in Henan. In the second batch of ten species, nine have been found in Henan: *Lantana camara* , *Ambrosia trifida* , *Pistia stratiotes* , *Solidago canadensis* , *Parthenium hysterophorus* , *Flaveria bidentis* , *Dysphania ambrosioides* , *Amaranthus spinosus* , and *Anredera cordifolia* . In the third batch, six of the ten species— *Amaranthus retroflexus* , *Symphyotrichum subulatum* , *Erigeron canadensis* , *Erigeron sumatrensis*, and *Erigeron annuus* , *Ipomoea purpurea* —are recorded in the province.

Our predictive modeling indicates that *Erigeron annuus* , *Conyza canadensis* , and *Ipomoea purpurea* have the largest potential suitable habitats in Henan, and all three species are included in the third batch of invasive plants, suggesting they may pose the greatest ecological threat to the region. *E. annuus* exhibits high adaptability and can establish in a variety of disturbed habitats (Guo, Wang, et al. 2024). *C. canadensis* is one of the most widespread invasive species in China and has strong invasion potential; it can inhibit the growth of neighboring plants through the release of allelopathic chemicals (Guo, Wang, et al. 2024; Wu, Zhang, et al. 2019). *I. purpurea* produces physically dormant seeds that can remain viable in soil for up to 17 years and has shown some resistance to herbicides (Wu, Zhang, et al. 2019).

Additionally, *Amaranthus spinosus* , *Erigeron sumatrensis*, and *Dysphania ambrosioides* exhibit the smallest potential distributions in Henan province. It is of particular importance to note that *A. spinosus* is currently registered as one of the most notorious IPSs in China, largely due to the significant threats it poses to the structure and ecological function of native ecosystems (Wang et al. 2024). *E. sumatrensis*, originally from South America, has spread widely across several Chinese provinces, including Guizhou, Hainan, Hunan, Jiangsu, and Jiangxi. Its invasive success is primarily due to its prolific production of tiny, lightweight seeds‐more than 20,000 per plant‐enabling easy dispersal by wind (Lan et al. 2024). *D. ambrosioides* and *A retroflexus* combine high fecundity, stress tolerance, and C4 photosynthetic efficiency—traits poised to exploit warming climates (Lin et al. 2025) *Ambrosia artemisiifolia* , due to its strong allergenic properties and wide distribution, is recognized by health authorities as one of the most hazardous invasive species in Europe (Wayne et al. 2002). The suitable habitat areas of *A. retroflexus* and *A. artemisiifolia* are projected to gradually decrease with future temperature increases. Notably, *Alternanthera philoxeroides* and *Bidens alba* are projected to expand under all scenarios (by 11.78%–24.44% and 1.85%–8.37%, respectively), suggesting that some highly adaptable species may benefit from warming regardless of emission trajectories. This finding underscores the need for species‐specific management strategies rather than uniform approaches. Given the severe invasiveness of these species, strengthened monitoring and management efforts are urgently needed to prevent further spread and minimize ecological damage.

Aquatic ecosystems are generally more vulnerable to alien plant invasions than terrestrial ones. For instance, *Alternanthera philoxeroides* , which can thrive in both aquatic and terrestrial habitats, is considered one of the most noxious invasive weeds in China and can exert negative impacts on the structure and function of native plant communities (Li and Denich 2024). *Eichhornia crassipes* thrives in aquatic environments and reproduces rapidly through vegetative propagation. Its invasion ability is largely attributed to the extensive clonal growth of stolons and free‐floating forms, with population doubling times as short as 1 week (Ren and Zhang 2007; Barrett 1992). *Pistia stratiotes* reproduces via vegetative buds or seeds, and its invasion often causes oxygen depletion at the water surface, inhibiting photosynthesis and increasing organic decomposition, which further lowers oxygen concentrations, potentially leading to local fish die‐offs and water eutrophication (Koleszár et al. 2024). In Henan, aquatic invasive species such as *A. philoxeroides* , *P. stratiotes* , and *E. crassipes* pose a significant threat to freshwater ecosystems if further spread occurs and thus warrant particular attention in future monitoring and control efforts.

Global warming has enabled alien species to expand into areas where they previously could not survive or reproduce (Walther et al. 2009). Most studies suggest that with ongoing climate change, the potential distribution range of invasive alien plants is likely to increase (Chu et al. 2024; Qin et al. 2024; Dullinger et al. 2017; Zhao et al. 2025). However, some species are projected to lose climatically suitable areas under future climate scenarios, particularly native species from northern and Mediterranean biomes (Dullinger et al. 2017). Our results indicate that by the 2050s, the suitable habitats for the most massive invasive alien plant in Henan Province are expected to contract. Currently, the combined potential suitable habitat of these species accounts for 18.42% of the total area of Henan. As greenhouse gas emissions rise, this area is projected to decline from 17.27% to 15.99% by 2050. These findings suggest that, overall, the situation regarding alien plant invasions in Henan may improve in the future. However, many regions will still face a high risk of invasion, particularly in high‐altitude areas.

Mountain ecosystems, especially those at high elevations, are among the few terrestrial ecosystems relatively unaffected by severe plant invasions, primarily due to harsh climatic conditions that can limit the spread of non‐native plants (Haider et al. 2011). However, with increasing residence time and ongoing climate change, alien species may expand their ranges and colonize new areas (Haider et al. 2010), potentially shifting from low to high elevations at an estimated rate of 11 m per decade as they track optimal climatic conditions (Chen et al. 2011). Our study shows that by 2050, the suitable habitats of invasive alien species will decrease in low‐elevation areas but expand into higher‐altitude mountain regions, a trend particularly evident in the mountainous areas of western Henan.

The observed unimodal pattern of massive invasive alien plants richness along the elevational gradient—increasing at low elevations, declining at mid‐elevations, and rising again at high elevations—can be explained by the interplay of environmental filtering, niche differentiation, and human disturbance gradients. The initial increase at low elevations likely reflects greater habitat heterogeneity and reduced extreme anthropogenic disturbance (e.g., intensive agriculture, urbanization) with slight elevation gains, consistent with Himalayan studies showing that both native and alien plant richness peak at similar elevations, suggesting that climatic productivity drives richness patterns for both groups (Rana et al. 2024). The subsequent decline at mid‐elevations aligns with the directional ecological filtering hypothesis (DEFH), whereby harsh climatic conditions (e.g., lower temperatures) and diminished propagule pressure progressively eliminate narrow‐niche species, retaining only broad‐tolerance generalists (Bacaro et al. 2015; Zhang et al. 2015); this pattern has been widely documented on Tenerife Island and in northern Chinese mountain forests (Bacaro et al. 2015; Zhang et al. 2015). The unexpected resurgence at high elevations (> 1500 m), while contrary to most regional studies (Bacaro et al. 2015; Zhang et al. 2015), may be attributed to the successful establishment of pre‐filtered generalist species that exploit novel mutualistic interactions at higher elevations—as observed in Argentina's Córdoba Mountains, where a single abundant seed disperser facilitated the invasion of a fleshy‐fruited shrub (Juncosa‐Polzella et al. 2023)—combined with the inherently subantarctic resistance of alpine communities, which, as demonstrated in subarctic mountain ecosystems, are more susceptible to invasion once propagules arrive via disturbed corridors such as roads (Williams et al. 2018).

Moreover, research indicates that most alien species are initially introduced at lower elevations, and as climatic conditions deteriorate and anthropogenic propagation pressure declines along elevation gradients, many are filtered out during the invasion process. However, some cold‐tolerant species may continue to spread into higher elevations (Freeman et al. 2018; Zhang et al. 2015), and once established, they may be difficult to control in alpine ecosystems with lower ecological resistance. In Henan, areas projected to become unsuitable for massive invasive plants may have already reached the ecological niche limits of these plants. Conversely, the upward expansion toward higher elevations poses a new threat to sensitive mountain ecosystems. Mountain tops are known to be among the most vulnerable ecosystems to climate change, and this trend may align with the “escalator to extinction” hypothesis (Freeman et al. 2018). As temperatures rise, species tend to migrate upslope, which can increase interspecific competition and potentially lead to the extinction of summit‐dwelling species already at their elevational limits. For the aforementioned high–richness areas, future efforts should focus on strengthening supervision and preventing further spread of invasive alien plants. In low–richness areas, early monitoring and rapid response mechanisms should be enhanced to prevent the further expansion and establishment of invasive species, thereby mitigating invasion risks at the source.

In summary, mountain summits often serve as vital habitats for rare and endangered species. Preventing the invasion of alien plants into high‐altitude areas, which could compromise ecological diversity, should be a top priority in current conservation efforts. In Henan Province, monitoring and management of invasive alien plants should focus particularly on mountainous and aquatic environments. Special attention should be given to high‐risk species through the establishment of robust early warning and rapid response systems to effectively safeguard regional ecological security and biodiversity.

## 5 Conclusion

In summary, our study provides the first systematic documentation and analysis of massive invasive alien plant species in Henan Province, focusing on their key characteristics including taxonomy, species composition, life forms, habitat types, origin and introduction pathways, as well as potential distribution patterns. We identified a total of 34 massive invasive alien plant species, primarily belonging to families such as Asteraceae and Amaranthaceae. The dominant life forms among these species are annual or biennial herbs and perennial herbs. Most of the massive invasive alien species in Henan are terrestrial, reproduce via seeds, originate mainly from the Americas, and have been introduced intentionally.

Spatially, these massive invasive plants are predominantly distributed in the western and southern parts of the province. Using the MaxEnt model and ArcGIS, we successfully predicted the potential distribution areas of 17 selected massive invasive species. Our analysis revealed that the current potential suitable habitat of these species covers approximately 18.42% of Henan's total area. Furthermore, projections for 2050 indicate that the suitable habitat areas for most species will decline to varying degrees, with a risk of upward expansion into higher‐elevation regions. Additionally, the centroid of the suitable habitat areas is projected to shift southwestward.

Our model was based on 2822 occurrence records and 39 environmental variables, including 19 bioclimatic, 3 topographic, 16 soil surface variables, and 1 human footprint variable. These modeling results provide valuable insights for future management and control of malignant invasive plant species in Henan. The ecological risks and potential threats posed by these species are alarming, emphasizing the urgent need for effective prevention and control measures.

Overall, our findings offer a scientific foundation for the identification, monitoring, and management of massive invasive alien plants in Henan Province. Given the projected spread of these species, it is crucial to enhance early warning systems and implement targeted regional prevention strategies to minimize their adverse impacts on the environment and economy.

## Author Contributions

**Hang Wang:** conceptualization (lead), data curation (lead), investigation (lead), methodology (equal). **Gaoxiang Cheng:** software (equal), visualization (equal), writing – original draft (equal). **Lvyuan Niu:** investigation (equal), resources (equal), writing – review and editing (equal). **Yizhen Shao:** data curation (equal), investigation (equal), software (equal). **Fengqin Liu:** formal analysis (equal), investigation (equal), supervision (equal). **Yun Chen:** conceptualization (equal), methodology (equal), resources (equal). **Xiangyu Tian:** conceptualization (equal), data curation (equal), formal analysis (equal), funding acquisition (equal), investigation (equal), methodology (equal), project administration (equal), resources (equal), software (equal), supervision (equal), validation (equal), visualization (equal), writing – original draft (equal), writing – review and editing (equal). **Zhiliang Yuan:** conceptualization (equal), data curation (equal), formal analysis (equal), funding acquisition (equal), investigation (equal), project administration (equal), supervision (equal), writing – review and editing (equal).

## Funding

This work was supported by the Survey on the Occurrence, Damage, and Spread Risk of Agricultural Invasive Alien Species (13220144); Agricultural Invasive Alien Species Key Survey and Monitoring Project (019240153); Natural Science Foundation of Henan Province (242300420484).

## Conflicts of Interest

The authors declare no conflicts of interest.

## Supporting information


**Table S1:** Characteristics of malicious invasive alien plants in Henan.
**Table S2:** Information of environmental variables.
**Table S3:** Environmental factor correlations.
**Table S4:** Species selected environmental variables for MaxEnt models.

## Data Availability

Provided as electronically Supporting Information.
